# Genome Sequence of *Trypanosoma cruzi* Strain Bug2148

**DOI:** 10.1128/genomeA.01497-17

**Published:** 2018-01-18

**Authors:** Francisco Callejas-Hernández, Núria Gironès, Manuel Fresno

**Affiliations:** a Centro de Biología Molecular Severo Ochoa, Consejo Superior de Investigaciones Científicas, Universidad Autónoma de Madrid, Cantoblanco, Madrid, Spain; b Instituto Sanitario de Investigación Princesa, Madrid, Spain

## Abstract

*Trypanosoma cruzi* belongs to the group of mitochondrion-containing eukaryotes and has a highly plastic genome, unusual gene organization, and complex mechanisms for gene expression (polycistronic transcription). We report here the genome sequence of strain Bug2148, the first genomic sequence belonging to cluster TcV, which has been related to vertical transmission.

## GENOME ANNOUNCEMENT

*Trypanosoma cruzi* is a highly polymorphic parasite that belongs to the *Kinetoplastidae* order and is the causative agent of Chagas disease, also known as American trypanosomiasis, a chronic illness and one of the most neglected tropical diseases (1). Chagas disease is endemic in Latin America, but due to the migration of infected people, this disease has been extended to countries that are nonendemic for the disease, such as those in the European Union, making Chagas disease a serious public health problem (2, 3). Seven to 10 million people are chronically infected with this disease, and 10,000 to 14,000 deaths per year are caused by it (4). There are thousands of different strains of this parasite, but in 2009, a classification based on the genetic structure was proposed, establishing the existence of six separate clusters or discrete typing units (DTUs), named TcI to TcVI (5).

The complete genome of *Trypanosoma cruzi*, predominantly described as diploid, has been predicted to be around 105 Mb in length, distributed across 20 to 46 chromosomes; however, the total genome size can vary extensively among strains even of the same DTU, mainly due to aneuploidies and variations in gene copy number (6–8). This complex genetic content has been related to evolution, genetic conservation, and variability processes, but its marked differential behavior in *in vitro* and *in vivo* models proposes also its relationship with infectivity and disease development (9, 10).

To date, there are available public genomes of some strains belonging to DTUs I, II, and VI. We have sequenced genomic DNA from metacyclic trypomastigotes cultured in Vero cells and RPMI medium supplemented with 5% fetal bovine serum (FBS) at 37°C; strain Bug2148, belonging to DTU TcV, was sequenced by Pacific Biosciences technology (8-kb to 15-kb read length) and assembled with HGAP version 3 (11), obtaining 55.22 Mb distributed in 934 contigs, with 68× coverage, corresponding to 100% of its haploid estimated genome. Contigs with coverage lower than 15× and without any predicted gene were filtered from the assemblies, and as was expected for this kinetoplastid, the G+C content was around 50% (51.63%). About 91% of its complete predicted genes showed BLASTN similarities to available *Trypanosoma cruzi* predicted genes (including hypothetical genes and pseudogenes [http://tritrypdb.org/tritrypdb/]), in agreement with previous results (12).

### Accession number(s).

The complete genome sequence of Bug2148 has been deposited in GenBank under accession number NMZN00000000.
